# P-1279. Five-year trend analysis of carbapenem-resistant Enterobacterales, including isolates carrying metallo-β-lactamase genes in United States, Europe and adjacent regions during 2020–2024

**DOI:** 10.1093/ofid/ofaf695.1469

**Published:** 2026-01-11

**Authors:** Mariana Castanheira, John Kimbrough, Zachary Kockler, Rodrigo E Mendes

**Affiliations:** Element, North Liberty, IA; Element Iowa City (JMI Laboratories), North Liberty, Iowa; Element Iowa City (JMI Laboratories), North Liberty, Iowa; Element Iowa City (JMI Laboratories), North Liberty, Iowa

## Abstract

**Background:**

Carbapenem-resistant Enterobacterales (CRE) isolates are an urgent human health treat. Different from serine-carbapenemase-producing isolates for which active antimicrobial agents are clinically available, metallo-β-lactamase (MBL)-producing organisms are still an unmet need with limited therapeutic choices in clinical use in most countries. We evaluated the prevalence of CREs and MBL-carrying isolates in a 5-year surveillance study in European (includes Israel and Turkey) and US hospitals.

**Figure:**
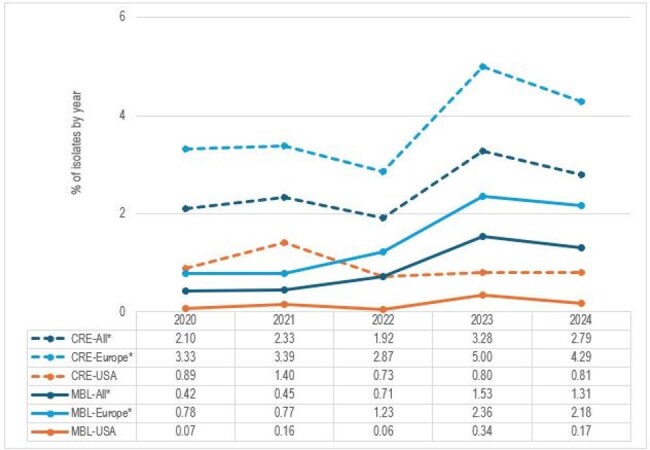
Percentage of CRE and MBL-producing isolates by year *Statistically significant trend (p<0.05)

**Methods:**

A total of 40,048 Enterobacterales were collected during 2020–2024 in 42 European and 37 US hospitals. Susceptibility (S) testing was performed by the CLSI reference broth microdilution method. CRE isolates (resistant to imipenem and/or meropenem using CLSI breakpoints) were submitted to whole genome sequencing and screened for MBL genes. Statistical analysis was performed using the chi-square test, with significance defined as *p*< 0.05.

**Results:**

Overall, 2.48% (994/40,048) isolates were CREs. CREs significantly increased from 2.10% (169/8,047) isolates in 2020 to 2.79% (223/7,995) in 2024 (*p*=0.002), but this increase was mostly noted in European hospitals where 3.33% (133/3,994) of the isolates from 2020 were CREs and these percentages were 4.28% (195/4,547) in 2024 (*p*=0.012) and 5.00% (235/4,704) in 2023. The numbers of CRE isolates in the US were considerably lower (28 isolates in 2020 and 36 in 2024) and yearly MBL rates were mostly < 1% without statistical significance (Graph). Among all CREs, MBLs were observed among 354 isolates. MBL carriers significantly increased from 0.42% (34/8,047) in 2020 to 1.31% (105/7,995) in 2024 (p< 0.001). These isolates significantly increased from 0.78% (31/3,994) in 2020 to 2.18% (99/4,547) in 2024 (p< 0.001) in Europe, whereas the numbers of MBL-carrying isolates in the USA were small (6 isolates in 2024 and 3 isolates in 2020).

**Conclusion:**

Monitoring MBL-carrying Enterobacterales isolates is an important endeavor to understand trends and potential spread of these isolates globally. In this evaluation of 5 years of a surveillance program, we observed a significant global increase in MBL-carriers that was mainly driven by an increase in MBL-carrying isolates from European countries.

**Disclosures:**

Mariana Castanheira, PhD, Melinta Therapeutics: Advisor/Consultant|Melinta Therapeutics: Grant/Research Support Rodrigo E. Mendes, PhD, GSK: Grant/Research Support|Shionogi & Co., Ltd.: Grant/Research Support|United States Food and Drug Administration: FDA Contract Number: 75F40123C00140

